# Simvastatin reduces melanoma progression in a murine model

**DOI:** 10.3892/ijo.2013.2126

**Published:** 2013-10-04

**Authors:** MARIO ZANFARDINO, CARMINE SPAMPANATO, ROSANNA DE CICCO, ELISABETTA BUOMMINO, ANNA DE FILIPPIS, SALVATORE BAIANO, ADRIANO BARRA, FRANCO MORELLI

**Affiliations:** 1 Institute of Genetics and Biophysics A. Buzzati Traverso, CNR Naples;; 2 Telethon Institute of Genetics and Medicine;; 3 Department of Experimental Medicine, Second University of Naples, Naples, Italy

**Keywords:** simvastatin, apoptosis, NonO, melanoma progression

## Abstract

Statins are a class of drugs that inhibit the rate-limiting step in the cholesterol biosynthetic pathway and show an anticancer effect, probably through the inhibition of cell proliferation. To date, the exact mechanism of cancer cell growth arrest induced by statins is not known. We report that simvastatin is able to induce apoptosis in melanoma cells but not in normal cells and also able to contrast the growth of tumor in an experimental melanoma murine model. We observed a delay in the tumor development in almost the 50% of the simvastatin administered animals and a strong reduction of the tumor volume with a differences of ∼150% compared to the controls. Also the survival rate was significantly higher in mice that received the drug with a survival increase of ∼130% compared to the controls. The tumor growth reduction in mice was supported by the results of cell migration assay, confirming that simvastatin clearly reduced cell migration. Moreover, simvastatin induced a strong downregulation of *NonO* gene expression, an important growth factor involved in the splicing regulation. This result could explain the decrease of melanoma cells proliferation, suggesting a possible action mechanism. The results derived from our experiments may sustain the many reports on the anticancer inhibitory property of statins and encourage new studies on this drug for a possible use in therapy, probably in combination with conventional chemotherapy.

## Introduction

Statins, the 3-hydroxy-3-methylglutaryl coenzyme A (HMGCoA) reductase inhibitors, are a class of drugs that inhibit the rate-limiting steps in the cholesterol biosynthetic pathway (1). Cholesterol is an important structural component of the cell membrane and the physiological requirements derive by endogenous synthesis or exogenous supply. Increases in lipid levels lead to atherosclerosis and narrowing of the blood vessels, which in turn may affect the blood supply to the heart, brain and peripheral circulation, leading to morbidity or mortality (2).

Statins, by inhibiting cholesterol biosynthesis, emerged as a principal agent in lowering the incidence of cardiovascular disease. However, it must be considered that any compound leading to depletion of cholesterol, which is the main structural component of cell membranes, affects various cellular events and impairs homeostasis. Statins, potent inhibitors of cholesterol synthesis, act by inhibiting 3-hydroxy-3-methylglutaryl CoA (HMG-CoA) reductase, which catalyzes the conversion of HMG-CoA to mevalonate (3). In addition to the cholesterol-lowering property, many biological effects of statins can be derived from cholesterol-independent pleiotropic mechanisms, which are likely a consequence of blocking intracellular signaling (4).

The role of statins extends beyond its lipid-lowering effects, as they are known to improve endothelial functions and participate in plaque stabilization, immune modulation and antioxidant activity and also acts as anti-inflammatory and anticancer agents. Their pleiotropic or cholesterol-independent effects at the cellular and molecular levels are highly related to numerous cellular functions, such as proliferation and differentiation. Treatment with simvastatin, mevastatin, atorvastatin, or pravastatin induces morphological change and decrease cell proliferation. It has been observed that the use of simvastatin was more effective in cancer cells and embryonic stem cells (ESCs), in relation to normal cells. In ESC, the loss of self-renewal by simvastatin was characterized by marked downregulation of several genes with function of ESC markers as alkaline phosphatase, Oct4, Nanog, Rex-1 and SSEA-1. Simvastatin effects were selectively reversed by either mevalonate or its metabolite, geranylgeranyl pyrophosphate (GGPP), but not by cholesterol or farnesyl pyrophosphate (5).

Besides their use in the treatment of lipid disorders, statins have been studied for their anti-carcinogenic effects in several models, including carcinomas of the colon and rectum, prostate, breast, lung and skin (6,7).

Many studies have shown the anti-proliferative and pro-apoptotic effects of statins to a greater degree both in malignant and in non-malignant cells (8,9). Statins can also trigger different tumor cells to undergo apoptosis *in vitro* and suppress tumor growth (10,11).

The role of cholesterol in cancer progression remains to be resolved but many tumor cell lines and tissues exhibit higher levels of cholesterol than their normal counterparts (12,13). Some reports indicate that hypocholesterolemia occurs in cancer due to increased use of cholesterol by tumors (14) whereas other reports have associated lower tissue cholesterol with malignancy (15). Epidemiological studies, the meta-analyses of statins use, and cancer risk in the general population have provided conflicting results. Some studies have shown cancer risk reduction associated with statins use (16–18) while other studies have reported no effect from its use (19–21) or even an increased risk (22). Unexpectedly, the typical response to simvastatin was greater in poorly-differentiated cells when compared to the well-differentiated cells (23).

In addition, substantial experimental and clinical evidence suggests that statins exhibit anticancer effects mediated by apoptosis and cell cycle arrest induction (24) through various signaling pathways. It has been hypothesized that statin-induced apoptosis is mediated by regulating BCL2 family members involved in mitochondrial apoptosis pathway of various cells types (1,9,11,25–28).

Interestingly, the same authors (29) found that p54^nrb^, a novel RNA binding protein with high homology to the PSF splicing factor has a high affinity for RNA via its N-terminus and can bind pre-mRNA and RNA implying a role in RNA processing (30,31). As a binding protein of single stranded RNA it mediates the splicing of several RNAs (32). Furthermore, p54 is known as a transcription factor activating the expression of several genes (33).

The nuclear p54^nrb^ protein, also called NonO (non-POU-domain-containing octamer binding protein), is an RNA-binding molecule of 54 kDa containing two RNA recognition motifs. p54^nrb^ is able to bind double-stranded DNA, single-stranded DNA and RNA, allowing the conclusion that p54^nrb^ has important roles in transcription and splicing (33). p54^nrb^ can either form heterodimers with protein-associated splicing factors or act as a monomer (32–39).

It may be speculated that p54 and *NonO* are needed for the proper expression of proteins leading to a stabilization of the transcription machinery, promoting the survival of cells (40,41).

The concerted expression of genes involved in different mechanisms of cell protection, like p54^nrb^ and NonO may contribute to the survival, promoting response counteracting the apoptosis in cancer cells.

Malignant melanoma is the most dangerous variant of skin cancer and prognosis of patients suffering from metastatic melanoma is poor. One of the key molecules regulating melanoma progression is the *protein melanoma inhibitory activity* (MIA) which is strongly expressed in malignant melanoma but absent in normal human melanocytes (42). Several studies suggest an important role for MIA also in the early tumor formation steps by regulating melanoma-related pathways and molecules (42). Recent investigations in mesenchymal stem cells hinted to protein p54^nrb^ as one of the MIA-regulated proteins (43). On these bases p54^nrb^ or the murine NonO seem to be a new molecule that function as regulator of malignant melanoma progression (44).

In this report we have investigated the role of simvastatin in the cancer cell growth inhibition showing the capacity of this drug to reduce cancer progression.

In the *in vivo* experiments, in which the melanoma was induced in C57Bl6 mice with the singenic B16-F10 melanoma cells, we observed an interesting reduction in the tumor volume and survival increase in the animals treated with simvastatin compared to the control group.

In addition, simvastatin induced a strong downregulation of NonO gene-expression, the murine homolog of p54^nrb^. On these bases the inhibited expression of NonO, in simvastatin treated cells, might suggest a possible action mechanism to explain the reduction of melanoma progression in mice administered with this drug.

## Materials and methods

### Ethics statement

All procedures were carried out in strict accordance with the recommendations in the Guide for the Care and Use of Laboratory Animals of the National Institutes of Health and mice were sacrificed using carbon dioxide (CO_2_) in accordance to the Guidelines for Humane Endpoints for Animals Used in Biomedical Research. Human endpoints were employed for the entire experimentation time and the mice showing signs of excessive distress or suffering were euthanized and eliminated from the experiments. We humanely sacrificed and eliminated from the experimentation any animals showing tumor with a diameter major >18 mm, that is the maximum tumor size recommended from the Guidelines of the Animal Experimentation Committee of IGB CNR, Naples, Italy. The study was approved by the Animal Experimentation Committee of IGB CNR, Naples, Italy.

### Cells culture

The B16-F10 mouse melanoma cells, NIH-3T3 mouse fibroblast cells, human melanoma SK-Mel-3 and the human melanoma A375 cells were purchased from American Type Culture Collection. All cells were grown at subconfluent culture in Dulbecco’s modified Eagle’s medium or RPMI supplemented with L-glutamine, 100 U/ml penicillin, 10 *μ*g/ml streptomycin and 10% fetal bovine serum, in 5% CO_2_ incubator at 37°C.

### Treatment with simvastatin

The simvastatin carboxylate form (Calbiochem-Merck Co., Darmstadt, Germany) is soluble in di-methyl-sulfoxide (DMSO) and at a minor rate in ethanol.

In our experiments, simvastatin was dissolved in DMSO prepared in a 20-mM stock solution stored frozen at −20°C. For the experiments, the cells were plated and treated with 20 *μ*M simvastatin for 48 or 24 h in normal culture conditions. At the end of treatment, cells were washed with PBS and used for TUNEL apoptosis analysis or harvested by scraping in TRIzol reagent (Invitrogen, Carlsbad, CA, USA) for mRNA expression analysis.

### TUNEL analysis

Apoptotic cells were detected with TUNEL using the Roche *in situ* cell death fluorescein detection kit (Roche Diagnostics Mannheim, Germany) following the manufacturer’s protocol. In brief, the plated cells were maintained in the presence of 20 *μ*M simvastatin for 48 h, after the treatment cells were washed with PBS, fixed with 2% PFA, permeabilized with 0.1% Triton X-100 in 0.1% sodium citrate solution, TUNEL reaction mix was added to the cells and incubated in a humidified chamber at 37°C for 60 min in the dark.

### RNA purification and cDNA synthesis

Total mRNA was extracted from control NIH-3T3 mouse fibroblast cells and simvastatin treated B16-F10 mouse melanoma cells, using TRIzol reagent (Invitrogen Co. Carlsbad, CA, USA) and the integrity of purified RNA was verified by agarose gel electrophoresis.

For cDNA synthesis 2 *μ*g of total RNA in a final volume of 25 *μ*l was reverse-transcribed with Avian myeloblastosis virus (AMV) reverse transcriptase (Gibco-BRL, Invitrogen), in the presence of random examer primers (Promega) at 37°C per 60 min, according to the manufacturer’s instructions. The cDNA was controlled by PCR with housekeeping GAPDH or actin primers.

### PCR analysis

Three different samples of the B16 melanoma cells and 3T3 control fibroblast were cultured for 24 h in DMEM containing 10% FCS and 20 *μ*M simvastatin. Control cells were cultured in identical culture conditions without simvastatin. Total RNA and cDNA synthesis was performed according the procedure described. PCR analysis of NonO gene expression was performed by using a GeneAmp PCR system 9700 (Applied Biosystem) and hot start Taq Gold (Applera). Mouse GAPDH or actin was used as a housekeeping control gene. The sequences of primers used were: mouse NonO Fw, TTA ACT TGG AGA AGC AGA ATC; mouse NonO Rv, CAG GCA AAG CGC ACT CGC AGC; actin Fw, GAC TAC CTC ATG AAG ATC CT; and actin Rv, GCT TGC TGA TCC ACA TCT GC; mouse GAPDH Fw, TCC CTC AAG ATT GTC AGC AA; mouse GAPDH Rv, AGA TCC ACA ACG GAT ACA TT. PCR conditions for mouse GAPDH and actin were: initial denaturation at 95°C for 10 min followed by 35 cycles: 95°C for 45 sec, 60°C for 45 sec and 72°C for 45 sec with a final extension at 72° for 10 min. For mouse NonO amplification the annealing temperature was 54°C and 42 PCR cycles. The amplification products were analyzed on agarose gel to control the amplicons length.

### B16-F10 cell wound healing assay

In order to understand the effect of simvastatin on progression and invasion of B16 cancer cells, we performed a wound-healing assay. In brief, cells were seeded onto 60-mm dishes at 5×10^5^ cells per plate. When the confluence reached 90%, a single scratch wound was created on the plate with a pipette yellow tip. After 24 and 48 h, in untreated cells or in presence of 20 *μ*M simvastatin, the cell capacity to grown through the scratch was verified. A quantitative analysis of cells migration was performed in the Boyden chamber.

### Boyden chamber cell migration assay

This analysis was carried out in a Boyden chamber under serum-free conditions. Polycarbonate filters, 10-*μ*m pore size, were coated with 5 *μ*g/ml fibronectin. After treatment with 20 *μ*M simvastatin for 24 and 48 h, 2×10^5^ B16-F10 cells were trypsinized and placed in the upper compartment of Boyden chambers in serum-free medium, while in the lower compartment, FBS was introduced as the chemoattractant. Cells were allowed to migrate for 4 h at 37°C in 5% CO_2_, fixed in ethanol and stained with haematoxylin. Ten random fields/filter were counted at ×200 magnification (45). In parallel, control cells were assessed for viability and counted using the trypan blue exclusion technique. Number of cells that had migrated was normalized to analyse the effects on cell viability.

### In vivo experiments

Animal experiments were performed in C57BL/6 mice of 20–25 g body weight, housed at 22–24°C under a 12-h light/dark cycle and with free access to water and food. The melanoma was induced in all C57BL6 mice by subcutaneus injection in the flank of syngenic B16 melanoma cells at a concentration of 1×10^5^ cells in 100 *μ*l PBS. All the animals were injected precisely with the same quantity of cells to avoid any difference in the tumor development derived from difference in cells inoculation. Drug-treated mice were administered, at alternate days, with simvastatin dissolved in DMSO at a concentration of 25 *μ*g/100 *μ*l DMSO intraperitoneally (simvastatin dose was 1 *μ*g/g body weight) while control animals were administered i.p. at alternate days with 100 *μ*l DMSO. The intraperitoneal administration was chosen to have a strong absorption and avoid differences in the treatment. The simvastatin administration started at the same time as the B16 cell injection. The tumor volume measures were made every 3 days starting after 10 days from cell injection (when the mass was measurable).

The experiment was conducted for 20 days before the animals died and the tumor became too large inducing animal sufferings. According to the Guidelines of the Animal Experimentation Committee of IGB CNR, we humanely sacrificed and eliminated from the experimentation any animals showing tumor with a diameter >18 mm.

The tumors diameters were measured using a caliper and tumor volume was calculate with the ellipsoid volume formula V = 4/3 πR_x_R_y_R_z_, that considering the small radius on the y-axis equal to the small radius on z-axis, were simplified in V = 1/6π (small diameter)2 (large diameter). In the experiment 22 mice were administered with simvastatin while 20 control mice received only DMSO.

### Survival rate

The survival analysis was conducted in accordance to the Guidelines for Humane Endpoints for Animals Used in Biomedical Research within an interval time of 18 days. To avoid animal suffering, we sacrificed all the mice when the tumor was >20 mm, that occur after 18 days from tumor onset. The mice were sacrificed using carbon dioxide (CO_2_). The number of dead mice was recorded every day and plotted on the Kaplan-Meier diagram.

### Statistical analysis

Tumor volumes during the growth were analyzed by Student’s t-test, and significance was set at p<0.05. For all the animal groups the mean and the standard error are reported (SEM).

## Results

### TUNEL analysis

**Figure 1.**
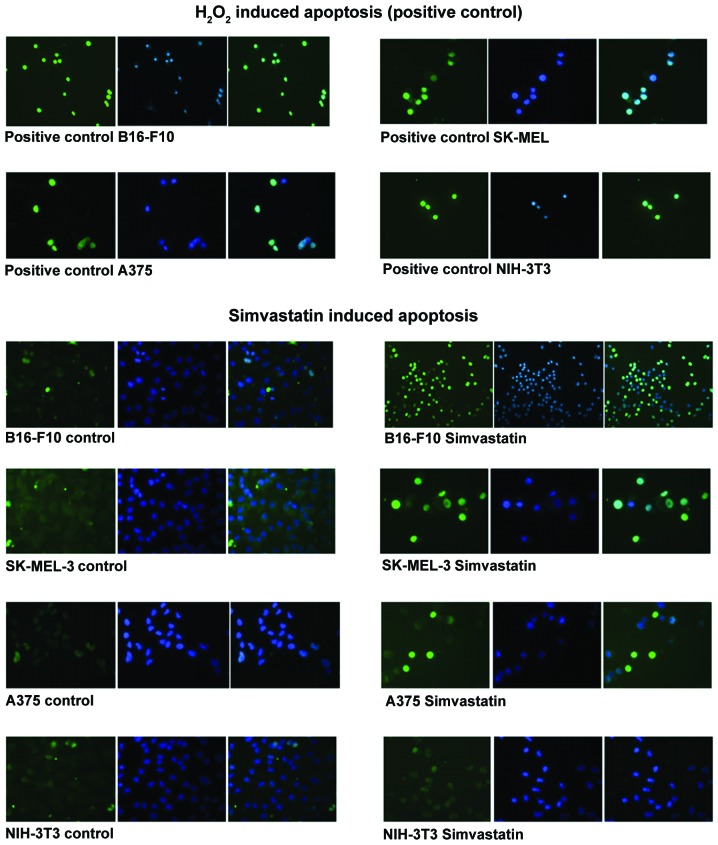
TUNEL analysis. Positive control of TUNEL reaction is obtained by inducing apoptosis with hydrogen peroxide in B16-F10 murine melanoma cells, in two lines of human melanoma the SK-Mel-3 and the A375 cells and in the NIH-3T3 mouse fibroblast. In all these cells the green fluorescein coloration inside the nuclei demonstrate clearly the apoptosis. In all the melanoma cells, both of murine or human origins, the simvastatin induces a strong apoptosis as indicated by the clear green fluorescence in the cells nuclei. On the contrary the non-cancer NIH-3T3 mouse fibroblast, used as control, appear to be resistant to simvastatin treatment. This experiment was repeated three times confirming the apoptosis induced by simvastatin in cancer cells but not in normal fibroblasts.

The TUNEL analysis clearly showed that 48-h treatment with 20 *μ*M simvastatin induced apoptosis in three different types of melanoma cancer cells, the mouse B16-F10 melanoma cells and the two human SK-Mel-3 and A375 melanoma cells. On the contrary, the non-cancer NIH-3T3 fibroblasts did not show apoptosis signs even when the drug treatment was prolonged >48 h. As positive control of TUNEL assay, apoptosis was induced by 30-min treatment with hydrogen peroxide. For all cell lines treated with simvastatin, the nuclei DAPI staining (blue) and the apoptotic nuclei (green) and the merge image were observed (Fig. 1).

The merge image clearly demonstrate that apoptotic signal (green) was localized only in the nuclei, showing that 48-h treatment with 20 *μ*M simvastatin induced strong apoptosis in cancer cells but not in non-transformed fibroblasts. In NIH-3T3 control cells, after 2 days of treatment, there was no apoptotic stain in the nuclei. On the contrary, apoptosis induction, with a strong green nuclear staining, was evident in B16 murine melanoma cells. Similar results were observed in the human melanoma SK-MEL and A375 cells. In all cancer cell lines, but principally in the human melanoma cells, there was a high number of non-viable cells after a 48-h drug treatment, and only some cells remained attached on the culture plate. In these residual cells, a very strong nuclei green coloration was observed due to apoptosis induction (Fig. 1). This experiment was performed three times confirming apoptosis induced by simvastatin in cancer cells, but not in normal fibroblasts.

### NonO expression in simvastatin treated cells

**Figure 2.**
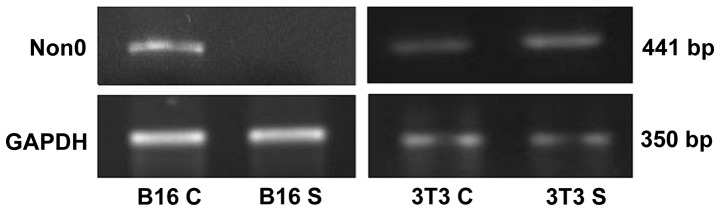
NonO PCR analysis. The NonO amplification in B16-F10 mouse melanoma cells and in NIH-3T3 mouse fibroblast treated (S) or non-treated (C) with 20 *μ*M simvastatin per 24 h. GAPDH is reported as internal control and demonstrates that 24-h simvastatin treatment did not damage the transcription apparatus. In this PCR analysis NonO was amplified for 42 cycles and GAPDH for 35 cycles. It is clear that simvastatin treatment inhibits the expression of NonO gene in melanoma cells but not in non-cancer fibroblasts.

In our experiments 24-h treatment with simvastatin completely inhibits the expression of NonO in melanoma cells (Fig. 2). After 24 h of simvastatin treatment there were no apparent signs of apoptosis and the downregulation in NonO expression may not be a consequence of the simvastatin induced cells damage or death. NonO is involved in mRNA transcription, in splicing and consequently in protein synthesis and it may play an important role in cell proliferation and probably also in B16 melanoma progression. As shown in Fig. 2, simvastatin completely switched off the NonO expression in B16-F10 melanoma cells, while the expression of this gene was present in untreated B16 cells. NonO expression is not visible in simvastatin treated cells also after 42 PCR cycles, while it was clearly expressed in control untreated cells. In normal non-cancer fibroblast, the simvastatin did not inhibit the NonO gene expression, in fact, its expression did not changed in simvastatin-treated NIH-3T3 cells (Fig. 2). These data, strongly suggest a possible action mechanism of this drug in the cancer cell growth inhibition, but further experimentation are required to confirm NonO as a simvastatin target.

### B16-F10 cell wound healing analysis

**Figure 3.**
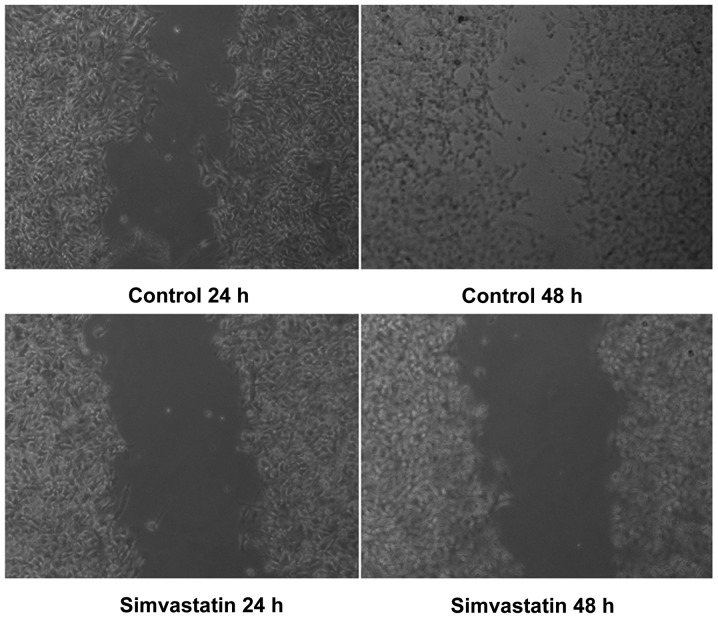
B16-F10 cell wound healing analysis. The simvastatin treatment clearly inhibits the B16 melanoma cell grown and migration. In untreated control cells after 48 h the scratch wound is repopulated, on the contrary in the plate administered with simvastatin, the cell growth is inhibited and the scratch remains clearly empty after 24 h and more significant also after 48 h in the culture, showing invasion inhibition. This experiment was replicated three times confirming the results obtained.

Simvastatin treatment clearly inhibited B16 melanoma cell growth after 24 and 48 h of culture in presence of the drug. Fig. 3 clearly shows that in the untreated cells the scratch wound is repopulated after 48 h, whereas in the plate administered with 20 *μ*M simvastatin, the cell growth is inhibited and the scratch remains empty after 24 and 48 h, demonstrating that simvastatin might inhibit cancer cells growth and migration. This experiment has been replicated three times confirming the results obtained.

### Boyden chamber cell migration assay

**Figure 4.**
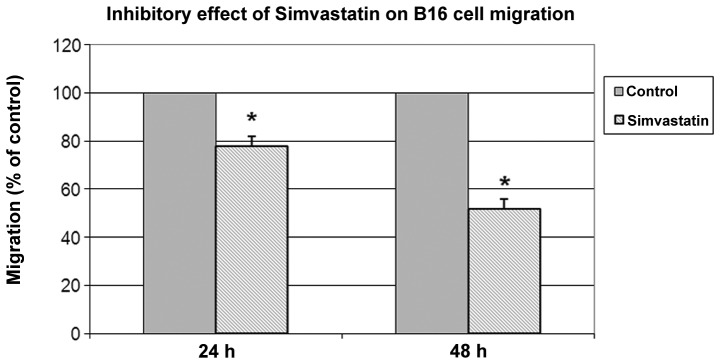
B16-F10 cells migration assay. The effect of simvastatin treatment on chemotactic migration of B16-F10 cells. Control and simvastatin-treated cells were left to migrate in presence of FBS. Data are reported as percentage of basal random migration in the presence of the chemoattractant. Results are reported as mean value of three different experiments. ^*^Significantly different compared to control (p<0.01).

To investigate if simvastatin was also associated with reduced cell invasion ability, a cell migration assay was performed. As shown in Fig. 4 simvastatin affected B16-F10 cell migration. After 24 and 48 h of treatment, cell migration inhibition, in comparison with untreated cells, was ∼22 and 48%, respectively.

### Tumor growth inhibition in simvastatin treated mice

**Figure 5.**
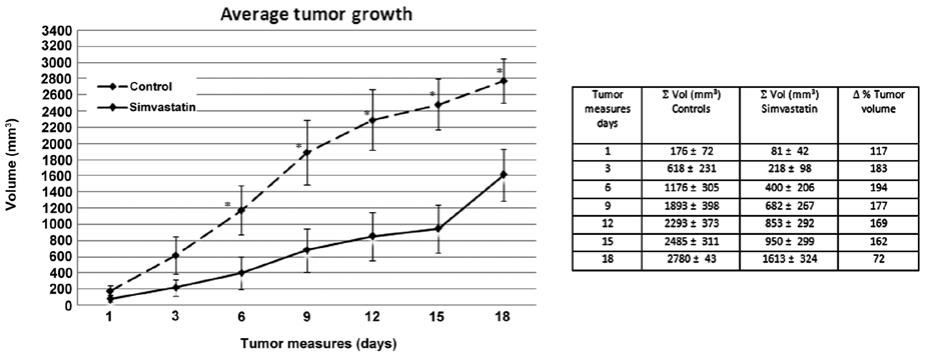
Simvastatin tumor growth inhibition. The average in tumor volume of simvastatin treated mice respect to the average size in the control group. The average volume values ± SEM and the difference (%) in tumor volumes, are reported in the table. Six days after the initial tumor measure, the average volume in the control group is ∼1200 mm^3^ but such levels were seen in the group treated with simvastatin only after >15 days. This denotes that simvastatin inhibits melanoma growth, resulting in an average <150% in simvastatin treated animals compared to the controls group. In the experiment 22 mice were administered with simvastatin while 20 control mice received only DMSO (^*^p<0.05).

To verify the hypothesis that simvastatin could limit melanoma growth *in vivo*, we administered the drug on B16 melanoma-bearing mice. Simvastatin at a dose of 1 *μ*g/g body weight dissolved in DMSO, was given intraperitoneally at alternate days for 20 days, starting the treatment contemporary with the tumor cell inoculation. The effect of this drug on tumor growth is shown in Fig. 5, in which the tumor volume reduction compared to the controls, is evident. Fig. 5 shows the average tumor size of simvastatin treated mice, compared to the average size in the control group during the treatment follow-up. We observed, already at the first tumor measure, 10 days after cells injection, a clear delay in tumor development in the simvastatin treated group. In these mice we observed a volume average of 81 mm^3^ whereas it was 176 mm^3^ in the control group. Furthermore, 6 days after the begin of tumor measures, the average tumor volume in the control group was ∼1200 mm^3^ compared to 950 mm^3^, the average volume reached in the group treated with simvastatin after >15 days of cancer development. These data, clearly demonstrate that in a murine *in vivo* model, simvastatin inhibits melanoma growth. In our experiment the tumor development, as a volume measure, is on average <150% in simvastatin treated animals compared to the control group for all the experimental time. The differences were significant with p-value ranging from 0.05 to <0.01.

### Survival rate curves

**Figure 6.**
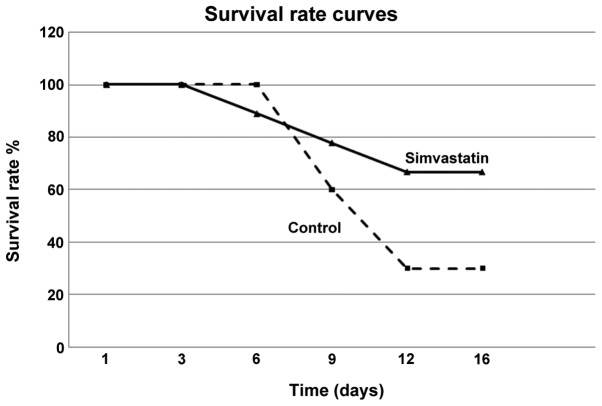
Survival curve analysis. Melanoma-bearing mouse survival in the presence of simvastatin treatment. In the untreated control group, 9 days after tumor onset, the survival was 60% and decreased to 30% after 12 days. On the contrary in the simvastatin treated animals, from day 9 to 16, the survival rate remained ∼70%. The survival rates were calculated daily and terminated at day 16. At day 18 all the mice were sacrificed.

To avoid animal suffering all the mice were observed in an interval of 18 days starting from tumor onset. The Kaplan-Meier curve, demonstrated that in the control group, after 9 days from tumor onset, the survival rate was 60%, and after 12 only 30%. On the contrary, in simvastatin treated animals, the survival rate remained ∼70% from day 9 to 16 (Fig. 6). These data confirmed that simvastatin treatment inhibited melanoma growth and progression in a well known murine model.

## Discussion

Many studies have shown that the role of statins extends beyond its lipid-lowering effects, as they are known to improve endothelial functions and antioxidant activity and also acts as anti-inflammatory and anticancer agents (46).

Their pleiotropic or cholesterol-independent effects at the cellular and molecular levels are highly related to numerous cellular functions, such as proliferation and differentiation. Treatment with simvastatin induced morphological change and decreased cell proliferation. It has been demonstrated that the use of simvastatin was more effective in cancer cells and embryonic stem cells (ESCs), in relation to normal cells (47).

Besides their use in the treatment of lipid disorders, statins have been studied for their anti-carcinogenic effects in several models, including carcinomas of the colon, rectum, prostate, breast, lung and skin (6,7).

Many studies have shown the anti-proliferative and proapoptotic effects of statins to a greater degree in malignant than in non-malignant cells (8,9). Statins also can promote different tumor cells to undergo apoptosis *in vitro* and to suppress tumor growth (10,11). The role of cholesterol in cancer progression remains to be cleared but various tumor cell lines and tissues exhibit higher levels of cholesterol than their normal counterparts (12,13). The splicing mechanism is an important point in the proliferation processes and all the factors involved in this regulation may be responsible for growth inhibition.

It may be speculated that either p54 or NonO are needed for the proper expression of proteins leading to a stabilization of the transcription machinery, protein production and function, promoting the survival of cells. The concerted expression of genes involved in different mechanisms of cell protection, like the human p54^nrb^ and the murine NonO may contribute to survival, promoting response counteracting the apoptosis in cancer cells. Our data suggest that either p54^nrb^ or NonO seem to be new molecules that function as regulator of progression of malignant melanoma cells (44).

In the present study we investigated the anti-proliferative effects of simvastatin on several types of melanoma cells and the inhibition of melanoma cell progression *in vivo*. In order to evaluate the growth inhibition induced by simvastatin treatment, we injected this drug at alternate days intraperitoneally. According to our results, the cancer cell growth inhibition is due to the deregulation of apoptosis induction and to the inhibition in the expression of the gene NonO. Our data suggest that simvastatin treatment might play an important role in inhibition of tumor progression, although more detailed studies are necessary to validate this hypothesis. Further experimentation is required to demonstrate if NonO is a common target of statin treatment and if the downregulation is common to other cancer cells apart from B16 melanoma cells and whether this effect is dose- and time-dependent.

In *in vivo* experiments, we demonstrated that simvastatin reduces tumor growth. In particular, we observed a delay in the tumor development in almost 50% of the animals treated with simvastatin respect to the control group. In these animals, for the first 10 days the tumor appeared very small, and in one of the simvastatin treated mouse, the tumor appeared after >20 days from cell inoculation whereas in the control mice and the majority of tumor-injected mice, tumors were evident after 10–12 days from the initial B16 cell injection.

The other very clear effect of simvastatin is the strong reduction in the tumor dimension as shown in Fig. 5.

Also the survival rate, as shown in Fig. 6, is significantly higher in mice that received simvastatin. In these animals after 12–16 days we recorded increased survival, that reached a difference of ∼150% respect to the controls.

The tumor growth reduction in mice is supported by the results of *in vitro* cell migration, as both by wound healing test, and by migration assay, simvastatin was shown to reduce cell migration.

The simvastatin ability to influence cell migration probably occurs by affecting integrin or downregulating the metalloproteinase production, two molecular effects that have been implicated in many steps of tumor dissemination. Also considering that metalloproteinase can be localized in a proteolytically active form on the surface of invasive melanoma cells (48). This may support the hypothesis of a possible function of the simvastatin in the metastasis inhibition.

Simvastatin induces a strong downregulation in the expression of NonO, the murine homolog of p54^nrb^. On these bases the inhibition of NonO, in cells treated with simvastatin, could explain the decrease of melanoma progression in mice administered with this drug, suggesting a possible action mechanism involving the NonO promoter, that may contain sequences with an high rate of mutation in cancer cells respect to the normal cells and that could function as a target for simvastatin. Further studies are necessary to verify whether NonO has an active role in cancer cell proliferation.

The results obtained may sustain the many reports on the anticancer inhibition property of statins and encourage the experimentation on this drug for the possible use in therapy probably in combination with the conventional chemotherapy.
